# Radiology Employees ‘Quality of Work Life in Tehran University of Medical Sciences Hospitals’ Radiology Departments

**DOI:** 10.5812/kmp.iranjradiol.17351065.3148

**Published:** 2011-11-25

**Authors:** Hussein Dargahi

**Affiliations:** 1 School of Allied Medical Sciences, Tehran University of Medical Sciences, Tehran, Iran

**Keywords:** Radiology, Iran, Work

Dear Editor,

In looking at Iranian healthcare organizations, one major problem is evident: “lack of strategic human resource management”. There may live several factors that result this lack of interest or know-how practice of Strategic Human Resource Management (SHRM).

Lack of SHRM in Iran might be the result of management definitions and methodologies that have been developed and used in Iran by delay because of their non-local origin. Strategic human resource has been used to formulate different models which have not considered uniqueness of the Iranian settings. It is well understood that human resource planning is a long term process that needs long term perspective and planning. The other side of this perspective exists in the current situation of Iran, a certain number of practitioners think that human resource planning is a short term operational program, not a significant power for their strategic plans. It is concluded that if there is a suitable strategy for human resource, designing systems, training, remuneration, promotion and employees’ development, an optimal profitability as well as an expert base for long term sustainability could be achieved. In the field of SHRM in Iran, its strategic function has not been considered and implemented by managers; it still is a personal management approach, dealing mainly with administrative functions. However, with the emergence of international co-operations and the entrance of multinational corporations, the need and importance of HRM is being felt more in Iranian healthcare organizations [1]. One of the most important HRM factors is the Quality of Work Life (QWL), an umbrella term including many concepts.

Consequently, concentrating merely on wages or management style, defined as job characteristics, is an inadequate approach for QWL assessment. Although job satisfaction is not QWL, perception of QWL is often assessed using job satisfaction surveys. Previous studies have shown that low job satisfaction is a major cause of turnover among healthcare providers [2].

A good employer knows the fact that employees have lives before and after work leading subsequently to creating trust and loyalty among employees, causing peacefulness for both employer and employee and a higher quality world [3]. Companies with high quality of work life can also enjoy exceptional growth and profitability [4].

In healthcare organizations, QWL has been described as referring to strengths and weaknesses in total work environment [5]. The attitudes and behaviors of healthcare workers have a crucial role on the delivery of high quality medical care requiring more analysis of employees programs designed for hospital employees [6]. Therefore, the work (task) performed and the supervision received by radiology directors (relationship with supervisor), the opportunity for career advancement, job security, adequate staffing and job stress as QWL factors are significantly related to job satisfaction and productivity [7][8].

QWL in Iranian Radiology Employees

A cross-sectional, descriptive study was conducted by the author among 170 radiology technologists and technicians in Radiology Departments of Teaching Hospitals of Tehran University of Medical Sciences (TUMS) from 2009 to 2010. Respondents were asked to express their attitudes about a range of key factors as the most important issues impacting their QWL.

The results of our research showed that most of the Iranian radiology employees were unsatisfied with their lack of job security, job environment and employees retention, career advancement, organizational reward system, job-service training, occupational health, especially radiation safety regulations in the radiology departments, lack of clear organizational goals and human relations considerations, job diversity, work overload, job assessment procedure and lack of their participation in organizational decision-making.

Implementation of SHRM for TUMS Radiology Employees

By considering the different levels of problems in implementing the human resource management area, the best recommended way is to apply the Excellence Model of European Foundation of Quality Management (EFQM) for the systematic implementation of SHRM in TUMS Radiology Departments. This model needs to cover different areas, while it is suited to the cultural characteristics of the country and organization.

Based on this excellence model, the result of such approach may be measured through the employees’ survey results. In this part, the organization management team will measure how effective their training is. If satisfying results are not obtained, new methods could be performed. For instance, considering an award or promotion for innovative, practical ideas. It seems the concept of HRM in Iranian healthcare organizations that have understood the importance of HRM is new, going back to four or five years. Currently, the trend is more toward strategic human resource management and there is hope in the future that the employees get the greatest benefit from their most valuable asset of their organization.

We believe that most of TUMS Radiology Department employees are dissatisfied with most of their QWL factors. Although we think low QWL is a huge problem in most countries, it is more unsatisfactory in Iran. We recommend that radiology care policy-makers and leaders should make substantial investments in the development of human resource management by preparing strategic planning based on strengths, weaknesses, opportunities and threats (SWOT) analysis and enhancing three model of EFQM in their sector.
